# Correlation Between Ischemia Time and Left Ventricular Failure After Primary Percutaneous Coronary Intervention in ST-Elevation Myocardial Infarction (STEMI) Patients

**DOI:** 10.7759/cureus.65268

**Published:** 2024-07-24

**Authors:** Sher Wali Khan, Ayesha Fayyaz, Ikram Ullah, Kainath Naeem, Hafsa Liaqat

**Affiliations:** 1 Cardiology, Lady Reading Hospital - Medical Teaching Institute, Peshawar, PAK; 2 Internal Medicine, Rheumatology and Allergy Institute of Connecticut, Manchester, USA

**Keywords:** predictors, primary percutaneous coronary intervention, stemi, left ventricular failure, ischemia time

## Abstract

Background: Primary percutaneous coronary intervention (PPCI) is pivotal in treating ST-elevation myocardial infarction (STEMI) patients, yet ischemia time significantly impacts outcomes, particularly left ventricular failure (LVF).

Objective: This study aimed to investigate the impact of ischemia duration and other variables associated with severe left ventricular systolic dysfunction in STEMI patients receiving PPCI treatment.

Methodology: This prospective cohort was carried out at Lady Reading Hospital in Peshawar, Pakistan, from January to June 2023. The study included 236 patients aged 18 to 70 with acute myocardial infarction who underwent PPCI within 12 hours of symptom onset. Patients with coronary dissection, late presenters (more than 12 hours after onset), those without stenting, and those with prior coronary artery intervention were excluded. Additionally, patients with systolic heart failure, a history of arrhythmias such as ventricular tachycardia or ventricular fibrillation, or a previous acute coronary syndrome event were excluded. Demographic information, clinical background, and ischemia duration were recorded and associated with left ventricular ejection fraction (LVEF) after PPCI. To identify predictors of severe left ventricular dysfunction, statistical analysis using SPSS Statistics version 26.0 (IBM Corp. Released 2019. IBM SPSS Statistics for Windows, Version 26.0. Armonk, NY: IBM Corp.) included multivariate regression, Pearson's correlation, and descriptive statistics.

Results: The patients' average age was 61.2 years (SD ± 12.3), with 35.59% of them being female (84 patients) and 64.41% of them being male (152 patients). Diabetes (33.05%, 78 patients) and hypertension (43.22%, 102 patients) were common comorbidities, and 14.41% (34 patients) had previously had a cardiac episode. Fifty-two patients (22.03%) of the total had ischemia within three hours, 94 patients (39.83%) had ischemia within six hours, 60 patients (25.42%) had ischemia within nine hours, and 30 patients (12.71%) had ischemia within 12 hours. Analysis of LVEF showed that 9.32% of patients (n=22) had LVEF <30% and 24.58% of patients (n=58) had LVEF 30-40%. Significant predictors of severe left ventricular systolic dysfunction were shown by multivariate regression to include ischemia duration (OR 1.45, p<0.001), age (OR 1.02, p=0.015), diabetes (OR 2.34, p=0.001), hypertension (OR 1.76, p=0.031), and previous cardiac events (OR 2.89, p=0.002); 20.33% of the patients (n=48) had LVF during the six-month follow-up, highlighting the therapeutic significance of prompt management in STEMI patients after PPCI.

Conclusion: Prolonged ischemia, advanced age, diabetes, hypertension, and previous cardiac events that predict severe left ventricular dysfunction are associated with a greater risk of LVF following PPCI. Timely intervention and thorough therapy are essential for enhancing results for STEMI patients at high risk.

## Introduction

Patients suffering from ST-elevation myocardial infarction (STEMI) should use primary percutaneous coronary intervention (PPCI) as the preferred reperfusion approach [1,2]. This method, involving mechanical revascularization of the occluded coronary artery, is highly effective in reducing myocardial damage, improving coronary perfusion, and enhancing clinical outcomes [3]. Even though PPCI is effective, its efficacy can be influenced by several factors, with a crucial aspect being the extent of myocardial damage and subsequent cardiac function, which is correlated with ischemia duration [4,5].

One of the most important factors influencing myocardial salvage is ischemia time, which is the amount of time between the start of symptoms of myocardial infarction and the restoration of blood flow in the afflicted coronary artery [6]. Extended periods of ischemia are linked to detrimental remodeling, a higher risk of heart failure, and widespread myocardial necrosis [7]. More specifically, among patients with STEMI, left ventricular failure (LVF) is a serious consequence that significantly affects deaths and morbidity [8]. Improving patient outcomes and treatment methods requires an understanding of the connection between ischemia duration and the emergence of LVF after PPCI [9].

The advantages of prompt PPCI in minimizing infarct size and enhancing left ventricular function have been well documented in the past [10,11]. The exact relationship between the length of ischemia and the degree of LVF is still unclear, however. Finding other risk factors for severe left ventricular systolic dysfunction in STEMI patients receiving PPCI is also of increasing interest [12,13]. The probability of developing LVF might also be influenced by variables such as the severity of myocardial infarction, other medical disorders, and the patient's clinical status upon admission [14,15].

The current state of research is lacking a thorough study that looks at other possible predictors of severe left ventricular systolic dysfunction in STEMI patients receiving PPCI in addition to quantifying the effect of ischemia duration on LVF. Closing this gap will increase our knowledge of the pathophysiological processes behind LVF and direct the creation of focused therapies to boost therapeutic results.

Research objective

The purpose of this research was to investigate the effects of additional markers of severe left ventricular systolic failure and the length of ischemia in STEMI patients undergoing PPCI.

## Materials and methods

Study design and settings

The prospective cohort research was carried out from January 2023 to June 2023 at the cardiology department of Lady Reading Hospital in Peshawar, Khyber Pakhtunkhwa, Pakistan.

Inclusion and exclusion criteria

The research included consecutive patients, aged 18 to 70 years, of both genders, who had acute myocardial infarction and had PPCI within 12 hours of the beginning of symptoms. Patients with coronary dissection, those with STEMI who presented more than 12 hours after the beginning of symptoms, those who had no stenting for whatever reason, and those who had previously undergone coronary artery intervention were all excluded.

Sample size

The sample size was calculated using the sample size formula by Pourhoseingholi et al. [16], taking into account the anticipated prevalence of coronary artery disease (p=19%).

Sample size (n) = z ² × p × (p-1) / d ² = (1.96)² × 0.19 × (1 - 0.19) / (0.05)² = 236

where d is the margin of error = 5% or 0.05 and z is the Z value from the standard normal distribution reflecting the confidence level, which in this case is 1.96 for a 95% confidence level.

Sampling technique

A non-probability consecutive sampling technique was used to recruit patients.

Data collection

Data on patient demographics, clinical presentation, and medical history were collected at admission, including age, gender, comorbid conditions (e.g., diabetes, hypertension), and prior cardiac events. The time of PPCI was used to record the ischemia time, which is the duration between the onset of myocardial infarction symptoms and the restoration of blood flow in the affected artery. Within 48 hours following PPCI, LVEF was used to measure the degree of left ventricular function; an LVEF of less than 40% was considered significant left ventricular systolic dysfunction. LVEF is an indication of left ventricular function, and a decrease in left ventricular function is closely associated with increased mortality. Left ventricular function was calculated using 2D echocardiography with multiple methods, including eyeballing, M-mode, and Simpson’s method, with an average of three readings taken. Following PPCI, patients were followed for six months to monitor the emergence of LVF and other adverse cardiac events.

Statistical analysis

SPSS Statistics version 26.0 (IBM Corp. Released 2019. IBM SPSS Statistics for Windows, Version 26.0. Armonk, NY: IBM Corp.) was used for data analysis. Descriptive statistics were used to summarize the baseline characteristics of the study population. Categorical variables were represented as percentages or frequencies, while continuous variables were represented as mean ± standard deviation (SD). The association between ischemia time and LVEF was evaluated using Pearson's correlation coefficient. Independent predictors of severe left ventricular systolic dysfunction were revealed by multivariate regression analysis, which took into account possible confounders such as age, gender, concomitant conditions, and infarct size. P-values less than 0.05 were regarded as statistically significant.

Ethical approval

The Institution Review Board of Lady Reading Hospital - Medical Teaching Institution issued approval 948/LRH/MTI. Every participant gave their informed permission, guaranteeing that the research complied with ethical guidelines and maintained patient privacy.

## Results

The baseline characteristics of the study population at Lady Reading Hospital in Peshawar, Pakistan, are presented in detail in Table 1. This includes the demographics and comorbidities of patients undergoing PPCI for STEMI. With a mean age of 61.2 years (SD ± 12.3), the distribution of patients by age group shows that 18 (7.63%) were older than 30 (12.71%), 44 (18.64%) were older than 41-50, 64 (27.12%) were older than 51-60, and 80 (33.90%) were older than 61-70 years. There were 84 female patients (35.59%) and 152 male patients (64.41%) in the research group. Comorbidities were common; 34 patients (14.41%) had a history of prior cardiac events, 102 patients (43.22%) had hypertension, and 78 patients (33.05%) had diabetes.

**Table 1:** Demographic and clinical characteristics of STEMI patients undergoing PPCI * Data on specific events was collected by asking patients to recall particular medical events and accessing their past medical records which are available on the HMIS electronic medical record system. STEMI: ST-elevation myocardial infarction, PPCI: primary percutaneous coronary intervention, HMIS: health management information systems

Characteristic		Number of patients (n)	Percentage (%)
Age groups (years)	18-30	18	7.63
	31-40	30	12.71
	41-50	44	18.64
	51-60	64	27.12
	61-70	80	33.90
	Mean ± SD	61.2 ± 12.3	
Gender	Male	152	64.41
	Female	84	35.59
Comorbid conditions	Diabetes	78	33.05
	Hypertension	102	43.22
Previous cardiac events*		34	14.41

**Figure 1:**
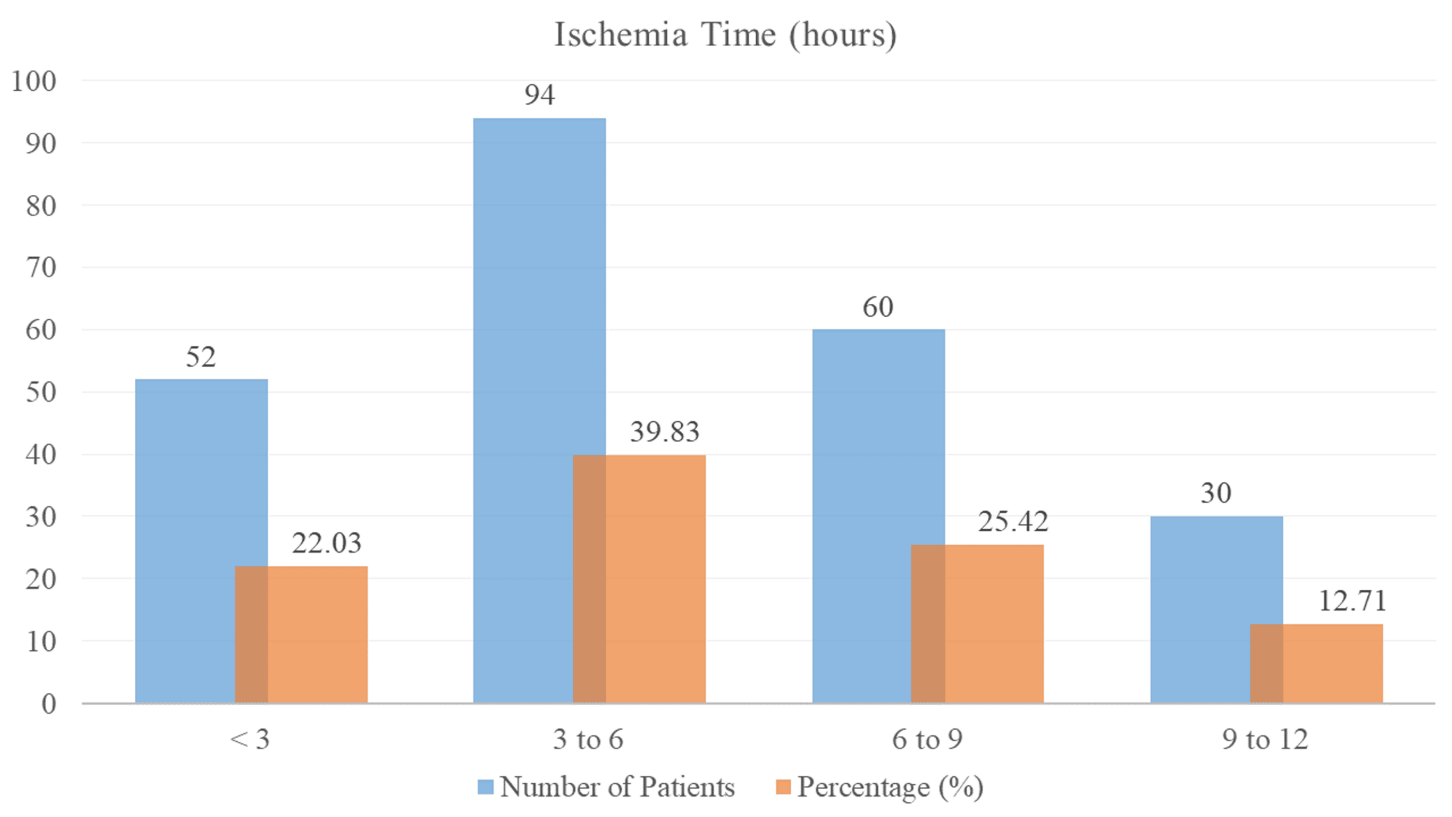
Distribution of ischemia time in STEMI patients after PPCI STEMI: ST-elevation myocardial infarction, PPCI: primary percutaneous coronary intervention

The distribution of ischemia duration in patients treated for STEMI with PPCI is shown in Figure 1. According to the statistics, 52 patients (22.03%) had ischemia for less than three hours, 94 patients (39.83%) experienced ischemia for three to six hours, 60 patients (25.42%) experienced ischemia for six to nine hours, and 30 patients (12.71%) experienced ischemia for nine to 12 hours. The different lengths of ischemia time that the research group experienced are reflected in these percentages, which is important for assessing how time to treatment affects cardiac outcomes in STEMI patients after PPCI.

**Figure 2:**
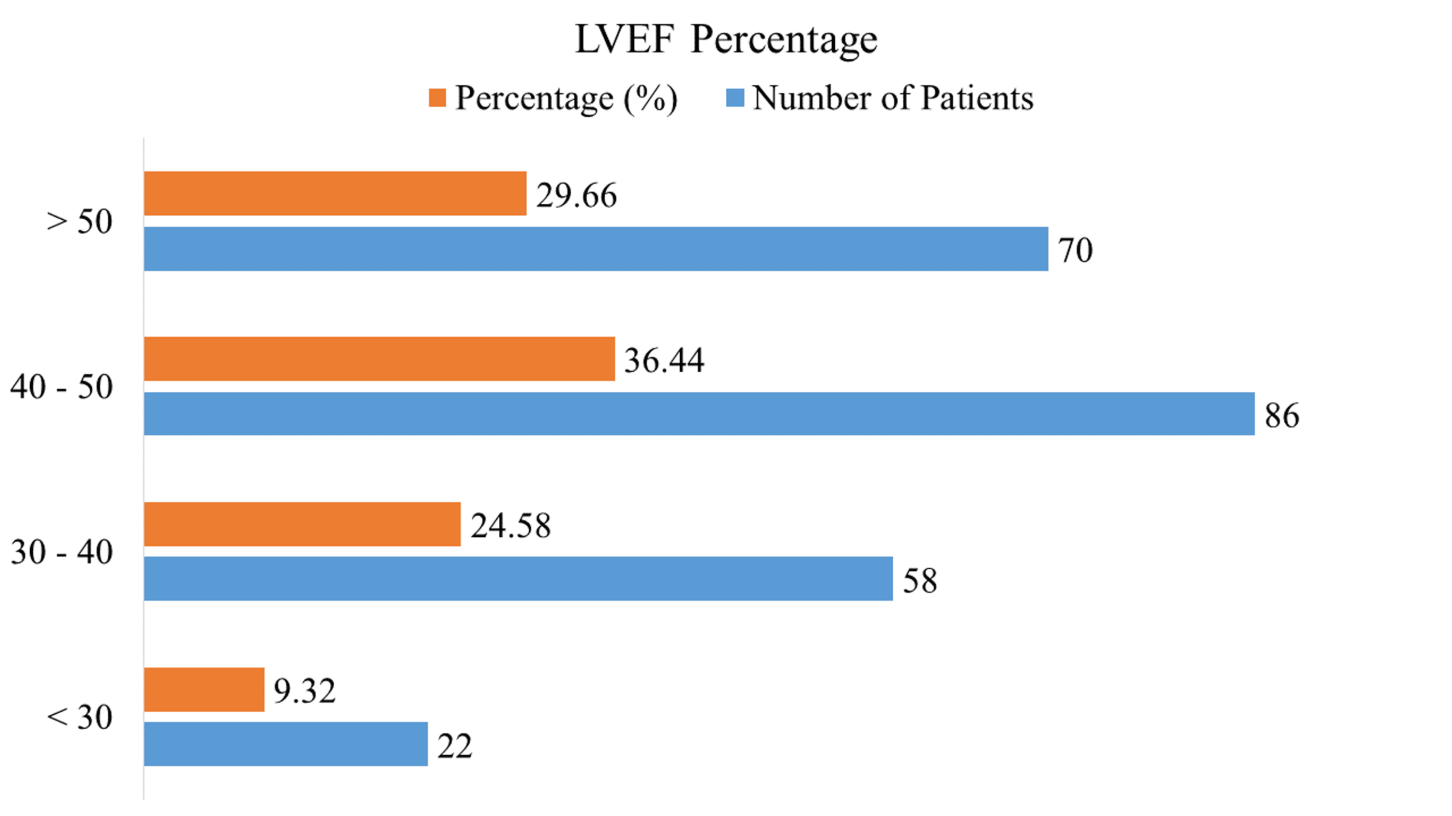
Distribution of LVEF in STEMI patients after PPCI LVEF: left ventricular ejection fraction, STEMI: ST-elevation myocardial infarction, PPCI: primary percutaneous coronary intervention

Figure 2 displays the distribution of LVEF in patients who had PPCI for STEMI. Fifty-eight patients (24.58%) had an LVEF between 30% and 40%, while 22 patients (9.32%) had an LVEF of less than 30%. Eighty-six patients (36.44%) of the total had a lower than 50% LVEF. Moreover, 70 patients (29.66%) had LVEFs greater than 50%. These percentages show how the LVEF categories were distributed throughout the study team, which is crucial information for assessing heart function in STEMI patients after PPCI.

Table 2 illustrates the correlation between the length of ischemia and LVEF in individuals with STEMI following PPCI. Given that there is a substantial negative connection (r = -0.62) between ischemia time and LVEF, it may be inferred that LVEF values are lower when ischemia durations are greater. The statistical significance of this relationship is demonstrated by the p-value below 0.001, which emphasizes the need for prompt reperfusion to maintain left ventricular function after PPCI.

**Table 2:** Correlation between ischemia duration and LVEF in STEMI patients

Variable	Correlation coefficient (r)	p-value
Ischemia time	-0.62	<0.001

The findings of multivariate regression analysis are shown in Table 3, which identifies predictors of severe left ventricular systolic dysfunction in patients with STEMI after PPCI. The study finds important correlations: a 1.45-fold increased risk of severe LV failure is related to every hourly upsurge in ischemia duration (OR 1.45, 95% CI 1.25-1.68, p<0.001). The risks of severe LVF are also substantially increased by older age (OR 1.02, 95% CI 1.01-1.04, p=0.015), diabetes (OR 2.34, 95% CI 1.40-3.91, p=0.001), hypertension (OR 1.76, 95% CI 1.05-2.97, p=0.031), and prior cardiac events (OR 2.89, 95% CI 1.50-5.55, p=0.002).

**Table 3:** Multivariate regression analysis of predictors of severe left ventricular systolic dysfunction OR: odds ratio, CI: confidence interval

Predictor	OR	95% CI	p-value
Ischemia time (hours)	1.45	1.25-1.68	<0.001
Age (years)	1.02	1.01-1.04	0.015
Diabetes	2.34	1.40-3.91	0.001
Hypertension	1.76	1.05-2.97	0.031
Previous cardiac events	2.89	1.50-5.55	0.002

Table 4 summarizes the incidence of LVF in patients with STEMI over a follow-up six-month period after PPCI. During the follow-up period, 188 patients (79.7%) did not have LVF, whereas 48 individuals (20.3%) out of the 236 patients under study did. This data emphasizes the significant percentage of patients who have LVF in spite of PPCI, highlighting the clinical importance of this condition and the need for efficient treatment techniques to enhance patient outcomes.

**Table 4:** Incidence of LVF in STEMI patients after PPCI LVF: left ventricular failure, STEMI: ST-elevation myocardial infarction, PPCI: primary percutaneous coronary intervention

LVF incidence	Number of patients	Percentage (%)
Developed LVF	48	20.33
Did not develop LVF	188	79.67

**Figure 3:**
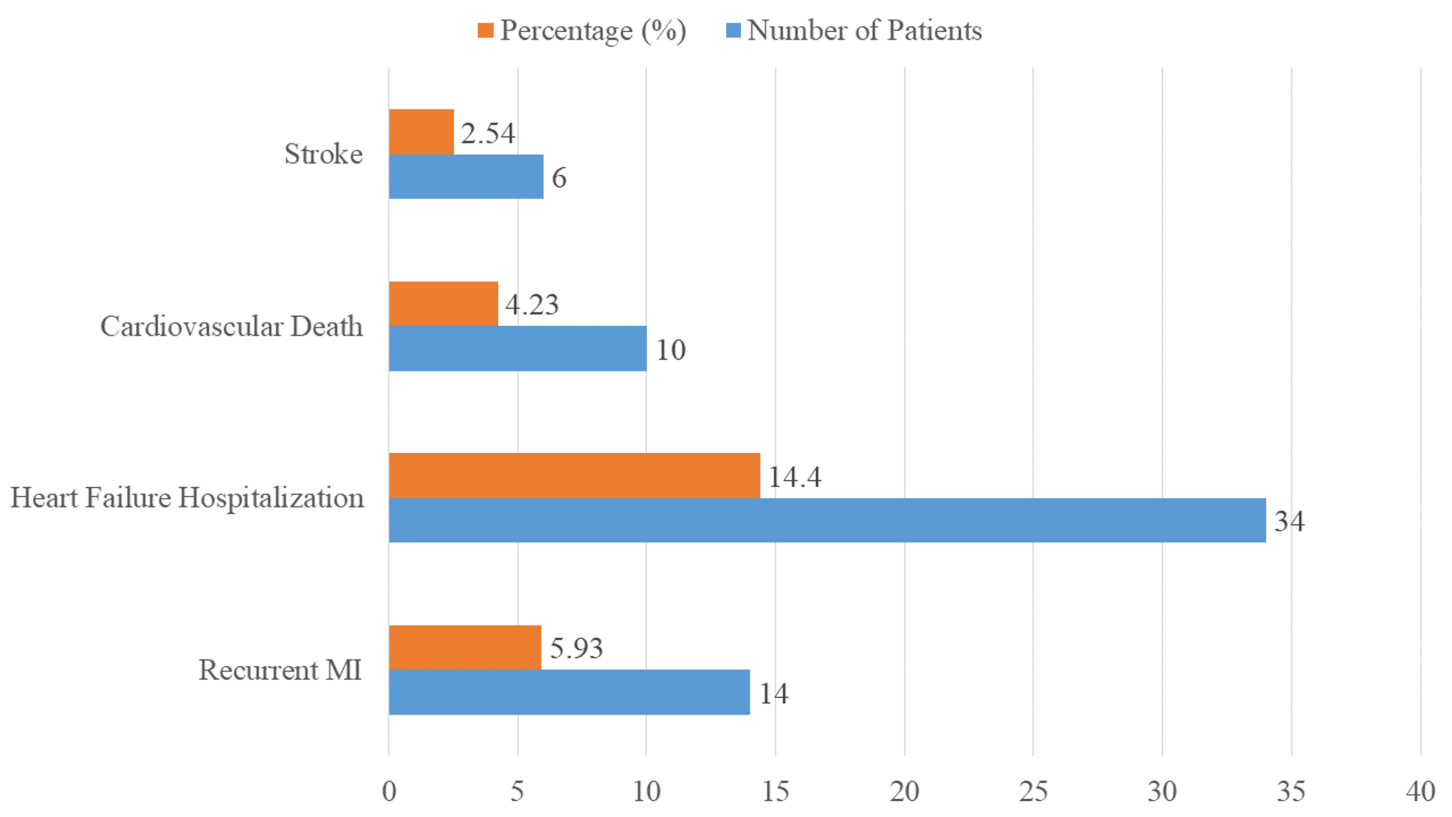
Adverse cardiac events during six-month follow-up in STEMI patients after PPCI STEMI: ST-elevation myocardial infarction, PPCI: primary percutaneous coronary intervention

The incidence of adverse cardiac events seen in 236 patients with STEMI over a six-month follow-up period after PPCI is shown in Figure 3. In particular, 34 patients (14.40%) needed hospitalization for heart failure, 10 patients (4.23%) died from cardiovascular causes, six patients (2.54%) had a stroke, and 14 patients (5.93%) had recurrent myocardial infarction.

## Discussion

Our research at Lady Reading Hospital examines the connection between left ventricular function and ischemia duration in patients receiving PPCI after STEMI. With a correlation value of -0.62 (p<0.001), we discovered a strong negative relationship between ischemia duration and LVEF. This strong connection shows that longer periods of ischemia are linked to worse outcomes for LVEF, underscoring the vital significance of timely reperfusion in preserving cardiac function and reducing myocardial damage. Our results are consistent with previous research demonstrating the detrimental effects of persistent ischemia on left ventricular function when compared to the body of literature now in publication. Research by Ndrepepa et al. [17] and Kiron et al. [18] also showed inverse relationships between ischemia duration and LVEF in STEMI patients receiving PPCI. When taken as a whole, these trials highlight how important prompt revascularization is for enhancing recovery and minimizing myocardial damage after STEMI.

Our multivariate regression analysis found many significant predictors of severe left ventricular systolic failure in addition to ischemia duration. An independent 1.02-fold increase in the probability of severe left ventricular dysfunction was linked to advanced age (OR 1.02, 95% CI 1.01-1.04, p=0.015). This result is consistent with a study by Josiah et al. [19], which similarly showed that age was a predictor of poor cardiac outcomes in patients with STEMI receiving PPCI. Age-related variables should be taken into consideration when developing care methods for elderly patients, as they may increase cardiac susceptibility and impair recovery after PPCI.

Furthermore, our analysis revealed that diabetes significantly predicted the development of severe left ventricular dysfunction, with an odds ratio of 2.34 (95% CI 1.40-3.91, p=0.001). This result is in line with that of Megaly et al. [20], who found that diabetes significantly increases the probability of unfavorable left ventricular outcomes in STEMI patients receiving PPCI. Otaal et al. [21] also had similar results to our findings and concluded that the presence of diabetes predicted a lower ejection fraction, a greater likelihood of developing LVF, and hemodynamic stability in patients presenting with anterior wall STEMI and total left anterior descending occlusion. The elevated risk seen in individuals with diabetes highlights the significance of glycemic control and all-encompassing cardiovascular risk management in reducing myocardial damage and enhancing therapeutic results.

Additionally, a significant predictor of severe left ventricular dysfunction was found to be hypertension, with an OR of 1.76 (95% CI 1.05-2.97, p=0.031). This is consistent with research by Reinstadler et al. [22], which similarly found that hypertension was a predictor of poor cardiac outcomes in individuals with STEMI who had PPCI. In hypertensive STEMI patients, optimal left ventricular function and a reduction in cardiac workload are critical for improving the long-term prognosis. These outcomes can only be achieved with effective blood pressure control.

Furthermore, with an OR of 2.89 (95% CI 1.50-5.55, p=0.002), our research demonstrated the prognostic importance of previous cardiac events in predicting left ventricular function after PPCI. This result supports that of Christopoulos et al. [23], who highlighted the cumulative effect of prior cardiac episodes on myocardial healing and resilience after revascularization. Understanding these variables may help with risk assessment and treatment plans targeted at improving outcomes for high-risk STEMI patients.

Strength and limitation

While current studies on PPCI in STEMI patients often lack a comprehensive evaluation of ischemia duration's impact on left ventricular function, our study addresses this gap by providing robust statistical analysis and prospective cohort data. This research strengthens the understanding of critical predictors, including ischemia duration, age, diabetes, hypertension, and previous cardiac events, underscoring the importance of timely intervention in improving outcomes for high-risk STEMI patients. However, our study has several limitations, including the use of non-probability sequential sampling, which may introduce selection bias, the exclusion of patients with late presentation or certain coronary conditions that might affect generalizability, and the relatively short follow-up period of six months, which may not fully capture the long-term outcomes of left ventricular dysfunction after PPCI. A significant limitation is the absence of the use of biomarkers, which have shown immense potential in predicting outcomes in STEMI patients. Many cost-effective and readily available biomarkers, such as NLR, FAR, and CRP, significantly aid in risk stratification and predicting the likelihood of patients developing LVF [24-26].

## Conclusions

Our research emphasizes how important ischemia duration is in predicting left ventricular function in patients receiving PPCI for STEMI. Prolonged ischemia, advanced age, diabetes, hypertension, and previous cardiac events that predict severe left ventricular dysfunction are associated with a greater risk of LVF following PPCI. Timely intervention and thorough therapy are essential for enhancing results for STEMI patients at high risk. A strong inverse relationship between extended periods of ischemia and LVEF was found, underscoring the deleterious effects of postponed reperfusion on cardiac consequences. In addition, severe left ventricular systolic dysfunction after PPCI was significantly predicted by advanced age, diabetes, hypertension, and past cardiac events. These results highlight the significance of prompt intervention and all-encompassing treatment approaches to maximize results and reduce cardiac damage in high-risk STEMI patients receiving PPCI. Future research should explore integrating biomarkers for early risk stratification, targeted therapies, and the use of technology for frequent, even remote, follow-ups, along with cardiac rehabilitation for all patients undergoing primary PCI.
